# Characterization of the complete chloroplast genome of *Populus deltoides* Zhonglin 2025

**DOI:** 10.1080/23802359.2020.1833773

**Published:** 2020-11-12

**Authors:** Weibing Zhuang, Xiaochun Shu, Ming Zhang, Tao Wang, Fengjiao Zhang, Ning Wang, Zhong Wang

**Affiliations:** a Jiangsu Key Laboratory for the Research and Utilization of Plant Resources, Institute of Botany, Jiangsu Province and Chinese Academy of Sciences (Nanjing Botanical Garden Mem. Sun Yat-Sen), Nanjing, PR China; b Jiangsu Forest Resources Inspect Center, Nanjing, PR China

**Keywords:** *Populus deltoides*, chloroplast genome, whole-genome sequencing, phylogenetic analysis

## Abstract

The complete chloroplast genome of *Populus deltoides* was characterized by reference-based assembly using whole-genome sequencing data. The total chloroplast genome size of *Populus deltoides* included a pair of inverted repeat regions (IRs) of 27,649 bp each, a small single-copy region (SSC) of 16,563 bp, and large single-copy region (LSC) of 85,096 bp, which was 156,957 bp in length. A total of 109 genes were predicted from the chloroplast genome, including 83 protein-coding genes, 22 *tRNA* genes, and four *rRNA* genes. The GC content of chloroplast genome for *Populus deltoides* was 36.68%. The phylogenetic analysis based on the reported chloroplast genomes of *Populus* showed that the chloroplast of the *Populus deltoides* is most closely related to the *Populus fremontii*. The complete chloroplast genome of *Populus deltoides* provides new insights into *Populus* evolutionary and genomic studies.

The *Populus* plants, naturally distributed in the Northern Hemisphere, are important economic species. In addition to reforestation, they can also provide raw materials for pulp, biofuel, and paper industries (Foster et al. 2015). *Populus deltoides* Zhonglin 2025 is a male plant with a well-developed root system, the rooting amount and root thickness of which are more than twice that of other poplars. As it does not produce catkins in spring to pollute the environment, it is an important tree species for urban beautification. In addition, the first color-leaf poplar (Zhonghong poplar) was obtained from a bud sport of *Populus deltoides* Zhonglin 2025 (Zhuang et al. 2018). Therefore, it is necessary to explore the complete chloroplast genome sequence of *Populus deltoides* to better understand its secrets.

The chloroplast is an important organelle in plants, which provides energy through photosynthesis and plays an important role in carbon uptake (Zhang et al. 2019). The chloroplast contains its own genome, and the genes of which are single-copy, avoiding the interference of side-line homologous genes. In addition, the genomes of chloroplast in higher plants are highly conserved in organization, gene order and content, which is important to explore the mechanisms of plant biology, diversity, evolution and climatic adaptation, and genetic engineering (Duan et al. 2020). With the development of high-throughput genome sequencing technology, it becomes much more convenient to acquire the complete cp genome sequences now than ever. Up to now, many chloroplast genome in *Populus* have been sequenced, while the complete chloroplast genome sequence is not available for *Populus deltoides*. In this study, the complete chloroplast genome sequence of *Populus deltoides* was characterized in order to further explore its physiological, molecular, and phylogenetical mechanism.

Fresh leaves of *Populus deltoides* Zhonglin 2025 were sampled from Nanjing Botanical Garden, Mem. Sun Yat-sen (E118_83, N32_06), Nanjing, China. The voucher specimen was deposited in the herbarium of Institute of Botany, Jiangsu Province, and Chinese Academy of Science under accession number of SAMN15594722. Genomic DNA was extracted using the DNeasy plant mini kit (Qiagen, Hilden, Germany). A paired-end library with an insert-size of 350-bp was constructed and sequenced on the Illumina NovaSeq system (Illumina, San Diego, CA). A total of 6030.9 Mb raw data were generated, and 5839.6 Mb clean data were used for the chloroplast genome reconstruction. The chloroplast sequence of *Populus trichocarpa* (NC_009143.1) was used as a reference (Tuskan et al. 2006). De novo genome assembly and annotation were conducted by NOVOPlasty (Dierckxsens et al. 2017) and GeSeq (Tillich et al. 2017), respectively. The raw sequencing reads used in this study were deposited in a public repository SRA, and the accession number is PRJNA660914. The annotated chloroplast genome was deposited in GenBank (accession number: MT789695).

The complete chloroplast genome sequence of *Populus deltoides* was 156,957 bp in length, with a large single-copy region (LSC) of 85,096 bp, a small single-copy region (SSC) of 16,563 bp, and a pair of inverted repeats (IR) regions of 27,649 bp. A total of 109 genes were annotated, including 83 protein-coding genes, 22 *tRNA* genes, and four *rRNA* genes. The GC content of the cp genome is 36.68%. To reveal the phylogenetic position of *Populus deltoides* with other members in *populus*, a phylogenetic analysis was performed based on 15 complete chloroplast genomes, and three taxa from *Salix* and *Vitis vinifera* were served as outgroups. The sequences were aligned by MAFFT version 7.309 (Katoh and Standley 2013). The maximum likelihood (ML) bootstrap analysis with 1000 replicates was performed using RaxML version 8.2.12 (Stamatakis 2014). The phylogenetic tree showed that *Populus deltoides* was closely related to *Populus fremontii* (Figure 1). The newly characterized *Populus deltoides* complete chloroplast genome will provide essential data for further study on the phylogeny and evolution of the genus *Populus* and of the *Salicaceae*, and provide useful resources for better understanding the physiology and evolution of the genus *Populus*.

**Figure 1.**
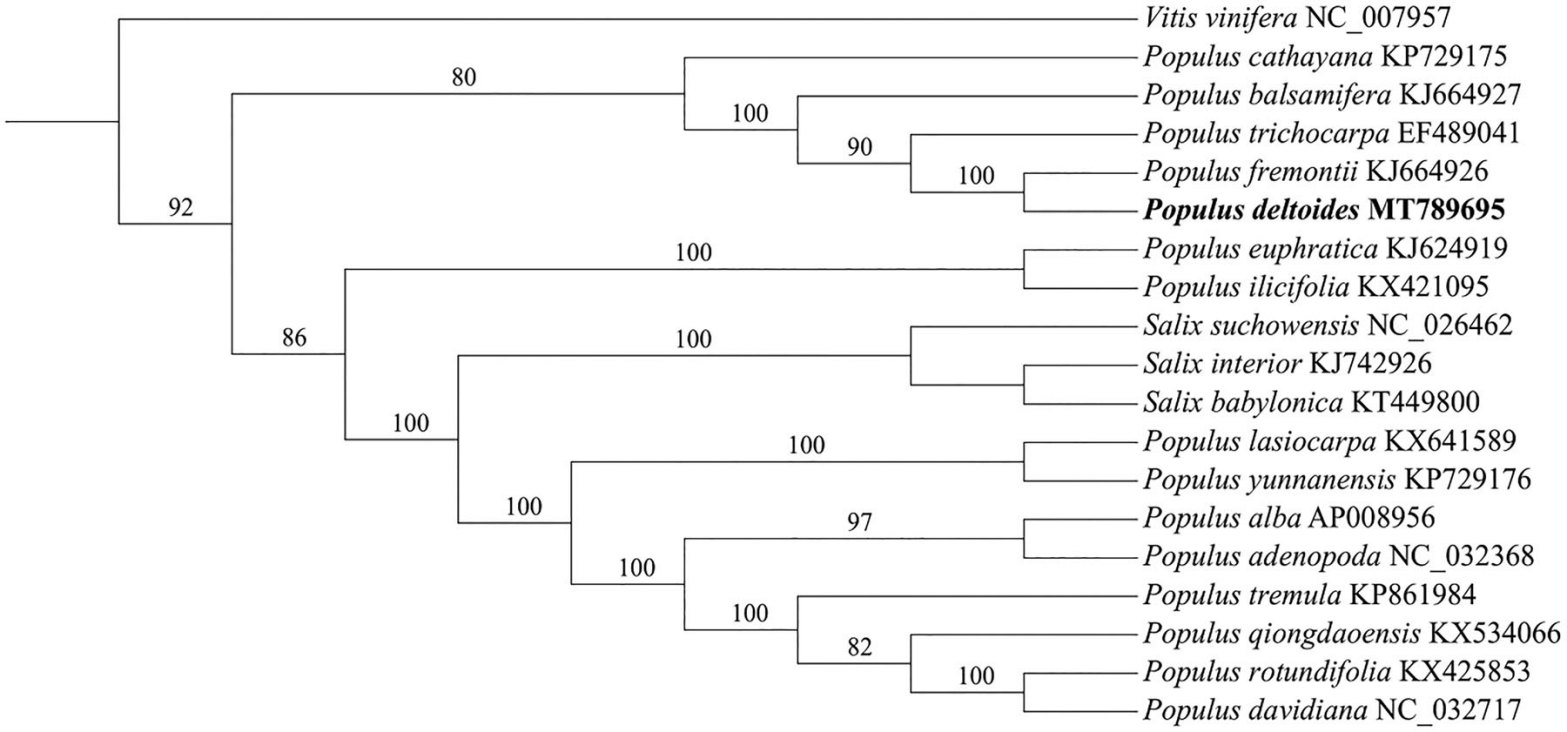
Phylogenetic tree was built with NJ methods using MEGA version 6.0 program based on 19 chloroplast genome sequences, and three taxa from Salix and Vitis vinifera were served as outgroups. Bootstrap support values (%) are indicated in each node. The accession numbers of chloroplast genome sequence for this tree construction are listed as follows: Populus alba (AP008956), *Populus adenopoda* (NC_032368), *Populus balsamifera* (KJ664927), *Populus cathayana* (KP729175), *Populus euphratica* (KJ624919), *Populus fremontii* (KJ664926), *Populus davidiana* (NC_032717), *Populus ilicifolia* (KX421095), *Populus qiongdaoensis* (KX534066), *Populus rotundifolia* (KX425853), *Populus trichocarpa* (EF489041), *Populus deltoids* (MT789695), *Populus yunnanensis* (KP729176), *Populus tremula* (KP861984), *Populus lasiocarpa* (KX641589), *Salix suchowensis* (NC_026462), *Salix interior* (KJ742926), *Salix babylonica* (KT449800), and *Vitis vinifera* (NC_007957).

## Data Availability

The data that support the findings of this study are openly available in GenBank of NCBI at https://www.ncbi.nlm.nih.gov, reference number MT789695.
